# Determinants of medication non-adherence among patients with chronic diseases at community pharmacy settings in South Gondar Zone, Northwest Ethiopia: a multicenter cross-sectional study

**DOI:** 10.3389/fpubh.2024.1409153

**Published:** 2024-10-18

**Authors:** Tilaye Arega Moges, Samuel Berihun Dagnew, Samuel Agegnew Wondm, Yared Andargie Ferede, Tesfagegn Gobezie Yiblet, Andargachew Almaw, Yohannes Shumet Yimer, Getu Tesfaw Addis, Woretaw Sisay Zewdu, Fisseha Nigussie Dagnew

**Affiliations:** ^1^Department of Pharmacy, College of Health Sciences, Debre Tabor University, Debre Tabor, Ethiopia; ^2^Department of Pharmacy, College of Health Sciences, Debre Markos University, Debre Markos, Ethiopia; ^3^Department of Medical Laboratory Science, College of Health Sciences, Debre Tabor University, Debre Tabor, Ethiopia

**Keywords:** medication non-adherence, chronic disease, determinants, pharmacy, patient safety

## Abstract

**Background:**

Medication non-adherence is a significant public health concern in managing patients with chronic diseases, and community pharmacists are on the frontline in the management of chronic medications. Chronic diseases require lifelong pharmacotherapy and understanding the determinants of medication adherence has paramount importance to develop strategies that improve medication adherence and treatment outcomes. Thus, this study aimed to assess the magnitude of medication non-adherence and its contributing factors among patients with chronic diseases attending community pharmacies in South Gondar Zone, Northwest Ethiopia.

**Methods:**

A multicenter cross-sectional study was conducted at community pharmacies in South Gondar Zone from September 01 to October 30, 2023. Medication adherence was assessed using a structured questionnaire using the Adherence in Chronic Diseases Scale (ACDS). Statistical Package for Social Sciences (SPSS) version 25 was used for analysis. Association between the outcome variable and independent variables was performed using binary logistic regression and a *p*-value of <0.05 was considered statistically significant.

**Results:**

Among three hundred and eighty six (386) study participants recruited in this study, more than half of them 222 (57.51%, 95%CI: 52.4% - 62.5%) were low adherent to their medications. Concerning determinants of medication non-adherence; the presence of side effects (AOR =2.1, 95%CI=1.33-3.29), unable to get ever counseling from community pharmacists (AOR=2.3, 95%CI= 1.46-3.58), and poor about their medications (AOR=3.1, 95% CI= 1.96-4.82) were significantly associated with patients’ non-adherence to medications.

**Conclusion:**

The medication adherence level in this study was suboptimal, with a significant proportion of the patients being non-adherent to their medications. The presence of side effects, unable to get ever counseling, and poor knowledge about their medications were statistically significant factors of poorer medication adherence. Hence, healthcare professionals, especially community pharmacists, have a crucial role in designing the schedule for health education concerning the needs of these patients in community pharmacy settings.

## Introduction

Chronic diseases are medical conditions that require long-term treatment for a lifetime (1, 2). They are often associated with high mortality and morbidity and are the leading causes of death globally, and annually, they account for 71% of all deaths. These include tuberculosis (TB), hypertension, and diabetes mellitus. Chronic disease prevalence is increasing worldwide. Their management requires an efficient and enforced healthcare system for ensuring quality and evidence-based healthcare services. Patients with chronic diseases require long-term maintenance use of medications (2–4).

Pharmacists in the community ensure medication safety throughout the medication-use process, and they provide a review of medications to ensure the appropriateness of treatment (5, 6). Community pharmacists play a critical role in patient safety since they have frequent contact with patients and hence ensure patients’ appropriate understanding of their medications (5, 7). This emphasized the development and implementation of effective management programs for patients with chronic disease at the primary healthcare level. One such intervention suggests that the involvement of community pharmacists in the management of chronic diseases has resulted in positive results in various healthcare settings in Ethiopia (8).

Medication non-adherence is associated with reduced treatment outcomes (9). If patients fail to get the expected health benefits because of medication non-adherence, the burden of healthcare in terms of high resource use and extravagant costs for patients and society might be increased (10, 11). Adherence to medications is an essential aspect of chronic disease management and is vital in achieving positive treatment outcomes (12). In 2003, the World Health Organization (WHO) identified that only 50% of patients with chronic diseases take their medication as prescribed in developed countries (13)).

To estimate medication adherence from pharmacy databases, the medication possession ratio (MPR) and the proportion of days covered (PDC) were commonly used. PDC is used by considering the proportion of days and a person’s medication access in a given period of importance (14, 15). Usually, for most medications, the threshold for adequate adherence has been placed at 80%, and for antiretrovirals, the threshold for adequate medication adherence has been placed at 80% (16). Improving health literacy (HL) may enhance the adherence of patients with chronic diseases, and HL intervention practice, monitoring, and evaluation for patients with chronic diseases are crucial to increase adherence to chronic medications and hence improve patient treatment outcomes (17).

Medication adherence is a key determinant in achieving therapeutic goals, and health education and proper medication counseling are necessary to attain medication adherence in patients with chronic diseases. Medication knowledge of patients can influence their medication use and adherence (18–20). Some recent studies revealed high rates of medication adherence; a systematic review among pre-dialysis chronic kidney disease (CKD) patients detected an adherence rate of 67.4% (21), and 76.44% of chronic patients were adherent to their medications according to a study in Tabuk (22). However, in several studies, medication adherence levels range from 38 to 57%, and approximately half of the chronic patients are non-adherent to their medications. Suboptimal treatment outcomes, high healthcare costs, adverse drug events, hospitalization, and frequent emergency visits are among the consequences of medication non-adherence (23).

In Ethiopia, some studies have been conducted on the medication adherence of patients with chronic diseases in public health institutions, focused on adherence to medications for specific diseases such as heart failure, diabetes mellitus, and/ or hypertension (24–31). In this study, we included patients with all types of chronic diseases, to make the results of this study more generalizable, and we conducted our study in community pharmacy settings since there is a paucity of previous studies regarding the magnitude of medication non-adherence on patients with chronic diseases in Ethiopia. Thus, this study aimed to assess medication adherence and factors associated with patients with chronic diseases at community pharmacies in South Gondar Zone, Northwest Ethiopia, using a structured questionnaire of the Adherence in Chronic Diseases Scale (ACDS). Therefore, this study has provided evidence-based results to improve patient care and help to fill gaps, which may contribute to enhancing chronic patients’ medication adherence and hence patient safety and treatment outcomes.

## Materials and methods

This multicenter cross-sectional study was conducted to assess determinants of adherence to chronic medications among patients with chronic diseases attending community pharmacies at South Gondar Zone, Northwest Ethiopia, from 1 September to 30 October 2023. South Gondar administrative zone is located approximately 667 km away from Addis Ababa, Ethiopia. According to the local report, the administrative zone has an estimated population of approximately 2,619,682 people. The seven towns in the South Gondar administrative zone, Northwest Ethiopia, in which community drug retail outlets (CDROs) were recruited in the present study include Debre Tabor, Gayint, Semada, Este, Addis Zemen, Andebet, and Woreta. As of October 2023, there were 134 active community drug retail outlets (CDROs) in the South Gondar administrative zone. Of these, 42 were pharmacies, and 92 were drug stores. Since the exact number of clients served in each community drug retail outlet is not known, we recruited an equal number of patients in each active drug retail outlet.

### Study population, inclusion, and exclusion criteria

Study participants were included in this study if they were adult patients (18 years or older) diagnosed with at least one chronic disease and patients with at least one medication for their chronic disease. Participants who were not willing to participate in the study, were critically ill to respond to the interview questions, or did not have a caregiver were excluded from the study.

### Sample size determination and sampling technique

The study participants were interviewed face-to-face by pharmacists at exit from the community pharmacy. A simple random sampling technique was to select study participants. The sample size was determined by using a single population proportion formula with the assumption of a 95% confidence level, 5% margin of error, and *p* = 60.7% which is the magnitude of medication non-adherence to chronic medications from the previous study (24). Therefore, the formula is *n* = [(Z1−*α*/2)^2^ * *p* * (1−*p*)] / d^2^, *n* = (1.96)^2^(0.607) (1–0.607) (0.05)^2^ = 367; by using contingency of 5%, that is, 367*5% = 19, the calculated sample size was 367 + 19 = 386, where d = margin of error, *p* = proportion of sample population, Za/_2_ = the value under a standard normal table using a 95% confidence interval, and *n* = the sample size.

### Study variables

The outcome variable consisted of medication adherence, while the explanatory variables consisted of socio-demographic parameters (gender, age, religion, current residence, marital status, education status, occupational status, medication fee, and monthly income) and clinical- and medication-related characteristics (number of medications per patient, comorbid disease, duration of chronic medication use, medication classes, medication regimen, counseling for all medications patient is taking, and the experience of side effects due to chronic medications).

### Data collection procedure and management

A structured interviewer-administered questionnaire that was pretested was designed based on previous studies conducted so far. A face-to-face, structured interview questionnaire was utilized by the three clinical pharmacists and two supervisors who had experience in data collection and research supervision concerning medication adherence, for patient face-to-face interviews to obtain demographic data, medication history, current medications, and their adherence to medications. The therapeutic group of medicines that a chronic patient is taking was classified according to the WHO Anatomical Therapeutic Chemical (ATC) Classification System. Among independent variables, medication knowledge was assessed by using tools adapted from previous studies (32, 33).

Patient knowledge was computed through a validated 7-item (yes/no) tool. A score of ≥5 is considered good knowledge, from a total of six questions, without question seven in considering the total score. The outcome variable, non-adherence to chronic medications, was assessed using the Adherence in Chronic Diseases Scale (ACDS) through self-reported adherence. The tool is available free of charge on the website of the Nicolaus Copernicus University in Poland (34). The multiple-choice equations include the following: 1. Do you always remember to take all your medications according to your doctor’s instructions? 2. Do you happen to change the dosing of your medications without prior consultation with your doctor? 3. Do you adjust the dosing of your medications according to how you feel? 4. On the appearance of medication-related side effects (e.g., stomach pain, liver pain, rash, lack of appetite, and edema), what do you do? 5. Do you find all your medications necessary for your health? 6. Does your doctor inquire about medication-related problems that you might experience? 7. Do you tell the truth when asked by your doctor about medication-related problems? The questions used for assessing medication adherence were translated into the Amharic language by experts in the area and back-translated to the English language before the pretest was conducted to minimize errors during translation.

Among seven questions of the medication adherence tool, the first five questions addressed the patient’s behavior related to the medication and the last two questions dealt with the relationship between patient and physician. The answer for each question was rated from 0 to 4 points. This makes the sum of the total adherence score range from 0 to 28. Medication adherence was labeled as high, medium, and low adherence if the total score was >26 points, 21–26 points, and <21 points, respectively.

### Data quality control

Before actual data collection, we conducted a pretest on 20 patients (5%) to ensure that the data collection tool was easily understood and accurately addressed the objectives of the present study, and those study participants were not included in the final data collection. It was only to ensure its clarity, uniformity, and understandability. During the pretest conducted, grammar errors, translation errors (from English to Amharic), and missed variables such as residence, presence of counseling service, and duration of chronic medication use were modified early before actual data collection. Training was given to data collectors, and daily supervision was carried out throughout the data collection period to ensure the accuracy and consistency of collected data on the adherence of patients with chronic disease care.

### Data analysis

The data were entered into the EpiData software (version 4.6.0.0) and analyzed using Statistical Package for Social Sciences (SPSS) version 25. Descriptive statistics was used to describe patient characteristics. Categorical variables were described using frequencies and/or percentages. The patient’s overall medication adherence was measured using the Adherence in Chronic Diseases Scale (ACDS), and it was labeled as high, medium, and low adherence if the total score was >26 points, 21–26 points, and <21 points, respectively. A binary outcome variable was generated by categorizing high and medium adherents as adherent (coded yes) and low adherents as non-adherent (coded no). A bivariate analysis was performed to identify determinants, and variables with a *p*-value of less than 0.25 were included in the multivariable analysis to control confounding factors. Multicollinearity was checked using variance inflation factor (VIF; all variables value less than 5) and tolerance test (above 0.2), and model goodness of fit was assessed by the Hosmer–Lemeshow test. Adjusted and crude odds ratios (ORs) with a 95% confidence interval were computed for each variable, and a *p*-value of less than 0.05 was statistically considered as significant.

### Ethical considerations

Ethics approval was obtained from the ethical review committee of the Debre Tabor University College of Health Sciences; Research & Community Service Coordination Office with the reference number DTU/CHS/335/2023, and both written and verbal informed consent were obtained from each study participants for data collection. Informed consent was obtained by clearly explaining the purpose and the procedures of the study and the right to withdraw from the oral interview was assured. The name of the study participant was not recorded; data were used only for the present study, respect was given to study participants, and involvement was based on volunteerism. During the training of data collectors, ethical issues were dealt with accordingly. All methods were performed according to the Declaration of Helsinki.

## Results

### Socio-demographic characteristics of the study participants

During the study period, 386 patients with chronic diseases were included and assessed for medication non-adherence at community pharmacies in South Gondar Zone, Northwest Ethiopia. More than half of them, 229 (59.3%), were male participants, and of the total participants, the majority (71.24%) were under 65 years of age. Concerning educational status, 146 (37.82%) of the participants had primary education, while 40 (10.36%) were unable to read and write (Table 1).

**Table 1:** Socio-demographic characteristics of study participants attending community pharmacy settings in South Gondar Zone, Northwest Ethiopia, from 1 September to 30 October 2023 (*n* = 386).

Characteristics	Total, *n* (%)	Adherent	Non-adherent
Gender			
Male participants	229 (59.3)	95	134
Female participants	157 (40.7)	69	88
Age (in years)			
<65	275 (71.24)	119	156
≥65	111 (28.76)	45	66
Residence			
Urban	162 (41.97)	72	90
Rural	224 (58.03)	92	132
Marital status			
Single	98 (25.39)	44	54
Married	260 (67.36)	110	150
Divorced/widowed	28 (7.25)	10	18
Educational status			
Cannot read and write	40 (10.36)	21	19
Non-formal education	41 (10.62)	12	29
Primary school (1–8)	146 (37.82)	62	84
Secondary school (9–12)	78 (20.21)	29	49
Tertiary education (diploma and above)	81 (20.98)	40	41
Job status			
Housewife	31 (8.03)	16	15
Farmer	143 (37.05)	57	86
Unemployed	65 (16.84)	31	34
Civil servant	57 (14.77)	26	31
Merchant	37 (9.59)	13	24
Other	53 (13.73)	21	32
Monthly income (Ethiopian birr)			
<1,500	120 (31.1)	43	77
1,500–4,000	103 (26.7)	48	55
4,000–5,000	78 (20.2)	31	47
>5,000	85 (22.0)	42	43
Presence of side effects of medicines			
Yes	180 (46.6)	57	123
No	206 (53.4)	107	99
Chronic medication counseling			
Yes	169 (43.8)	91	78
No	217 (56.2)	73	144
Good medication knowledge			
Yes	195 (50.5)	105	90
No	191 (49.5)	59	132
Number of medications consumed			
1–4 medicines	172 (44.6)	66	106
≥5 medicines	214 (55.4)	98	116
Duration of medication use			
<1 year	128 (33.2)	47	81
≥1 year	258 (66.8)	117	141

### Clinical- and medication-related characteristics of participants

Among 386 study participants recruited in this study, 204 (52.85%) of chronic patients consumed less than five medicines during their visits. The majority, 297 (76.94%), of patients with chronic diseases used the medication for a greater than 1-year duration (Table 1). The most frequently used medications based on their WHO ATC classification were for the cardiovascular system, 120 (31.09%), nervous system, 80 (20.73%), and anti-infective, 78 (20.21%; Table 2). The most frequent attending reasons were related to cardiovascular, 222 (57.5%), gastrointestinal, 159 (41.2%), and infectious, 132 (34.2%), diseases (Table 3).

**Table 2:** Medications used based on their WHO ATC classification by study participants attending community pharmacy settings in South Gondar Zone, Northwest Ethiopia, from 1 September to 30 October 2023 (n = 386).

Medication category by WHO ATC classification system	Frequency (%)
A: alimentary tract and metabolism	50 (13)
B: blood and blood-forming organs	12 (3.11)
C: cardiovascular system	120 (31.09)
D: dermatologicals	23 (5.96)
G: genitourinary system and sex hormones	20 (5.18)
H: systemic hormonal preparations, excluding sex hormones and insulin	12 (3.11)
J: anti-infective for systemic use	78 (20.21)
L: antineoplastic and immune-modulating agents	10 (2.59)
M: muscular-skeletal system	19 (4.92)
N: nervous system	80 (20.73)
P: anti-parasitic products, insecticides, and repellents	22 (5.7)
R: respiratory system	55 (14.25)
S: sensory organs	8 (2.07)
V: various	5 (1.3)

**Table 3:** Reasons (diseases detected during visit) for study participants to attend community pharmacy settings in South Gondar Zone, Northwest Ethiopia, from 1 September to 30 October 2023 (*n* = 386).

Reasons for attending community pharmacy	Frequency (%)^*^
Respiratory diseases	129 (33.4)
Infectious diseases	132 (34.2)
Gastrointestinal diseases	159 (41.2)
Cardiovascular diseases	222 (57.5)
Malnutrition diseases	14 (3.6)
Hematological malignancies	5 (1.3)
Musculoskeletal and joint diseases	50 (13)
Endocrine disorder diseases	39 (10.1)

* Cumulative percentage exceeds 100% since more than one disease happened for a patient.

### Medication adherence status of patients with chronic diseases

**Figure 1:**
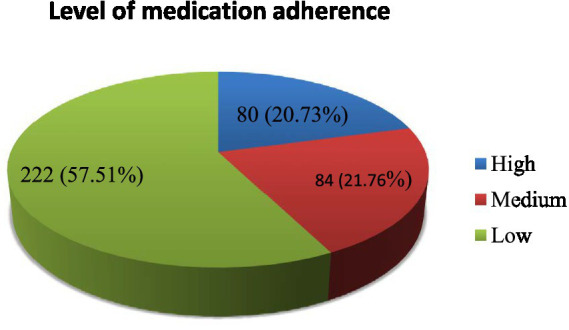
Medication adherence of patients with chronic diseases attending community pharmacies in South Gondar Zone, Northwest Ethiopia from 1 September to 30 October 2023.

The majority of study participants, 222 (57.51%), at 95% CI (52.4–62.5%) were low-adherent to their medications (Figure 1).

### Factors associated with non-adherence to chronic medications

As shown in Table 4, analysis of the data with multivariable logistic regression revealed associations between the level of knowledge and adherence among chronic disease patients at community pharmacy settings in South Gondar Zone, Northwest Ethiopia. Chronic patients with poor knowledge about their medications are significantly associated with medication non-adherence in this study. In addition, the presence of side effects and not getting chronic medication counseling were found to be significantly associated with patients’ non-adherence to chronic medications. Patients with poor medication knowledge had 3.1 times higher odds of being non-adherent than those with good medication knowledge (AOR = 3.1, 95% CI: 1.96–4.82, *p* < 0.00). Patients who experienced side effects had 2.1 times higher odds of being non-adherent than those without side effects (AOR = 2.1, 95% CI: 1.33–3.29, *p* < 0.01). Patients who did not receive chronic medication counseling had 2.29 times higher odds of being non-adherent compared to those who received counseling (AOR = 2.29, 95% CI: 1.46–3.58, *p* < 0.00). In the present study, the number of medications dispensed (1–4 vs. ≥5) was not significantly associated with medication non-adherence (AOR = 0.73, 95% CI: 0.46–1.14, *p* = 0.17). Monthly income was not found to have a statistically significant association with patients’ non-adherence to chronic medications. There was no statistically significant association between the duration of medication use (<1 vs. ≥1 year) and medication non-adherence (AOR = 0.78, 95% CI: 0.49–1.26, *p* = 0.31; Table 4).

**Table 4:** Bivariate and multivariable logistic regression analysis of factors associated with chronic patients’ medication non-adherence at community pharmacy settings in South Gondar Zone, Northwest Ethiopia (*n* = 386).

Variables	Category	Adherent, *n* (%)	Non-adherent, *n* (%)	COR (95% CI)	AOR (95% CI)	*p*-value
Good medication knowledge	Yes	105 (27.2)	90 (23.32)	1	1	
	No	59 (15.3)	132 (34.2)	2.6 (1.72,3.96)	3.1 (1.96, 4.82)	0.00^*^
Monthly income (Ethiopian birr)	<1,500	43 (11.14)	77 (19.95)	1.75 (0.99, 3.08)	1.73 (0.94, 3.21)	0.09
	1,500–4,000	48 (12.44)	55 (14.25)	1.12 (0.63, 1.99)	1.33 (0.71, 2.48)	0.38
	4,000–5,000	31 (8.03)	47 (12.18)	1.48 (0.79, 2.76)	1.36 (0.69, 2.66)	0.37
	>5,000	42 (10.9)	43 (11.14)	1	1	
Presence of side effects	Yes	57 (14.77)	123 (31.87)	2.33 (1.54, 3.54)	2.1 (1.33, 3.29)	0.01^*^
	No	107 (27.7)	99 (25.65)	1	1	
Number of medications	1–4 medicines	66 (17.1)	106 (27.5)	1	1	
	≥5 medicines	98 (25.4)	116 (30.0)	0.74 (0.49, 1.11)	0.73 (0.46,1.14)	0.17
Chronic medication counseling	Yes	91 (23.58)	78 (20.2)	1	1	
	No	73 (18.9)	144 (37.3)	2.3 (1.52, 3.48)	2.29 (1.46, 3.58)	0.00^*^
Duration of medication use	<1 year	47 (12.2)	81 (21.0)	1	1	
	≥1 year	117 (30.3)	141 (36.5)	0.7 (0.45, 1.08)	0.78 (0.49, 1.26)	0.31

1 = a reference used. * = *p*-value < 0.05 in the multivariable analysis which is considered significant.

## Discussion

Community pharmacists are on the frontline in the management of chronic diseases, and pharmacist-led medication adherence intervention was effective at improving medication adherence and clinical outcomes in patients suffering from chronic diseases (35, 36). The objective of this study was to assess determinants of medication adherence among patients with chronic diseases at community pharmacy settings in South Gondar Zone, Northwest Ethiopia, and this has paramount importance to clinical practice. Medication non-adherence is a public health concern as it contributes to poor treatment outcomes, reduced quality of life, and high healthcare costs (11, 37). Medication adherence in chronic disease patients is relatively suboptimal because those patients, mostly after the first 6 months of medication consumption, fail to persist in taking the medications as intended and usually drop their medications unexpectedly (38).

Addressing the issue of medication non-adherence is among the top healthcare policies and research agendas for stakeholders, and medication non-adherence is associated with poor outcomes in patients with chronic diseases (39, 40). The findings of the current study showed that the majority, 222 (57.5%), at 95% CI (52.4–62.5%) of the patients were low-adherent to their medications. This finding was consistent with the study among patients with chronic diseases in Italy and among adult chronic disease patients who were taking oral medications attending community pharmacies in Gondar, Ethiopia, where 60.7% of patients were low-adherent to their medications (24, 30, 41).

The findings of the present study were lower than those of the study conducted in Tanzania, where approximately 313 (74.7%) patients had poor adherence (42), and a study in Nigeria in which the majority of the patients were non-adherence to their medication treatment (71.2%) (43). The findings of this study were higher than those of the study in Saudi Arabia, and 22.9% of study participants had poor adherence to medications (44); in a study in Jordan, 47% had poor medication adherence (45); and in a study in Jordan, 36.2% of participants were low-adherent to medications (46). The difference in the magnitude of medication non-adherence may be due to a slight variation in the study population, study area, data collection tool, operational definitions, measurement tools of medication adherence, and level of care in both developing and developed countries across the world.

Pharmacists should have their role in patient education and counseling by using adherence aids, especially for those patients with low literacy rates in this study (47). Pharmacists can increase medication adherence of patients with chronic diseases by using motivational communication skills, reviewing patients’ medication regimens, supervising treatment efficacy and safety, and discussing with other healthcare providers the management of drug-related problems such as missed dose, route, frequency, or duration of therapy (48). Health literacy (HL) has a positive impact on chronic disease management (49) and has its role in improving patient counseling by healthcare professionals, and inaccessibility of written information for patient education on medications and related issues may affect chronic patients’ adherence diseases on chronic medications (50, 51).

Identified factors associated with medication non-adherence provide healthcare professionals (HCPs) with important health information to ensure medication safety and ultimately patient safety. Those patients who report the presence of medication side effects are more likely to be non-adherent to their medications than those patients who report no side effects (AOR =2.1, 95% CI = 1.33–3.29, *p* = 0.01). This result is in line with the study in Jeddah, Saudi Arabia (44). Patients who do not get ever counseling on medications from community pharmacists have increased odds of medication non-adherence to their medications (AOR =2.3, 95% CI = 1.46–3.58, *p* = 0.00). This result is consistent with the studies that revealed that patient ever counseling had a good impact on improving medication adherence (52, 53).

After possible adjustment for confounders, poor medication knowledge was significantly associated with non-adherence in the current study. Knowledge of patients on medications is one of the most important components of chronic disease management among patients with chronic diseases (32, 33, 54). The findings of our result pointed out that poor knowledge of chronic patients about their medications was one of the factors associated with medication non-adherence, implying that patients with good knowledge have a high level of medication adherence. Chronic patients with poor knowledge had increased odds of non-adherence to their medications (AOR = 3.1, 95% CI: 1.96–4.82, *p* = 0.00). Poor medication knowledge was three times more likely to have medication non-adherence than to those chronic patients with good medication knowledge. This study finding is consistent with studies conducted previously (55, 56). This might be due to those patients with good knowledge having a better understanding of the management of the disease, the advantages and importance of adherence, and the consequences of medication non-adherence (57).

This strong association between level of knowledge and medication adherence implies the need for continuous health education to improve patients’ awareness about their medications and the nature of the disease, especially in community pharmacy settings. Healthcare workers should pay special attention to establishing a strong relationship with patients to increase knowledge regarding determinants of adherence, complications, comorbidities, and management of chronic diseases. Patients struggle to reconcile daily life with comorbidity, and multiple medications may be poorly understood (58). Patient-centered care requires a greater understanding of the daily decisions patients need to make to manage a complex medication regimen.

Since medication non-adherence is a serious issue in managing patients with chronic diseases (38), pharmacists in community settings should proactively participate in proper medication counseling and patient education about the rational use of medications with persuasive communication skills. If community pharmacists fail to do so, medication non-adherence might happen and lead to poor patient treatment outcomes and higher treatment costs (11). Therefore, job training about chronic medication management and communication skills should be provided to the healthcare professionals (most importantly primary care physicians and community pharmacists) who provide services for patients with chronic diseases.

### Limitations and strengths of the study

The first limitation is linked to study design, whereby claims about the directionality of causal relationships cannot be verified between the outcome and independent variables in a cross-sectional study. Second, face-to-face interviews may increase the inclination of study participants to socially acceptable answers rather than the actual ones. The strength of this study is that it did not assess not only medication adherence but also its relationship to various determinant factors. Moreover, the response rate was 100%; all patients approached were involved in the study, providing a better representation of the target population with chronic diseases. Future national-based longitudinal studies could address these potential limitations by using larger, more representative samples, incorporating more detailed medication-related and clinical-related factors, and employing more robust medication adherence measurement techniques.

## Conclusion and recommendations

The medication adherence level in this study was suboptimal, with a significant proportion of the patients being non-adherent to their medications. In this study, the presence of drug side effects, poor medication knowledge, and unable to get ever counseling from community pharmacists were found to be statistically significant factors associated with chronic patients’ non-adherence to medications. Hence, an intervention targeting the development of guidelines that improve the care of health facilities regarding the values of counseling and follow-up of chronic patients is highly demanded.

The findings of this study should be used as baseline information for healthcare policymakers, concerned governmental bodies, and stakeholders that work around rational medicine use. Healthcare professionals should design educational programs, such as enhancing communication skills between patients, primary care physicians, and clinical pharmacists, which improve the patient’s adherence to their medications, especially in patients with chronic diseases. Our results also provide evidence of the positive role of pharmacists on patients’ medication adherence in community pharmacy settings. Therefore, special attention should be given to the improvement of medication adherence since low medication adherence is a widespread issue across the world and can lead to more comorbidity, complications, and even death.

## Data Availability

The original contributions presented in the study are included in the article/Supplementary material, further inquiries can be directed to the corresponding author.
