# Supplementary material for: Determinants of medication non-adherence among patients with chronic diseases at community pharmacy settings in South Gondar Zone, Northwest Ethiopia: a multicenter cross-sectional study
Source: Front Public Health. 2024 Oct 18;12:1409153. doi: 10.3389/fpubh.2024.1409153 (PMC11527677; doi:10.3389/fpubh.2024.1409153)
Supplement: Supplementary file 1 [file Data_Sheet_1.PDF]

Supporting information

Annex I: Participant information sheet, and consent

Participants' information sheet

Name of principal investigator: **Tilaye Arega Moges**

Name of study area: Community pharmacy settings in South Gondar Zone, Northwest Ethiopia

Research budget covered by: There was no funding for this research

Research objective: To assess determinants of medication non-adherence among patients with c hronic diseases at community pharmacy settings in South Gondar, Northwest Ethiopia

Significance of the study: The study specifically targets the chronic disease management and adherence to chronic medications of patients with chronic diseases that require long-term treatment for a lifetime and are often associated with high mortality and morbidity, and the leading cause of death globally. Ensuring that medication adherence to chronic medications is crucial for improving patient treatment outcomes. Thus, this study has the potential to significantly impact the management of patients with chronic diseases and their adherence to medications, ultimately leading to improved patient care and health outcomes.

Risks: The risk could be the lowest to the patient during the study. The only threat for the interviewee could be spending time (may be a maximum of 30 minutes was spent).

Participant right: The right of interviewees to withdraw at any time from the interview or not to participate and to escape questions which are not comfortable for them was reserved.

Beneficial: The study could be beneficial for patients, health policy makers, and quality service delivery system to come across and to promote an intervention which enhance medication adherence for the future perspective.

Confidentialities: The study result did not include patients' name and address. Hence confidentiality was kept as data was accessed only by ethically conducted, well trained and experienced data collectors of the study as well as interviewed and analyzed by the principal investigator.

Agreement: Patients were fully voluntary to participate in the study, those who were not able to participate withdrawn early.

Whom to contact: If participants have any kind of difficulties about the study, fell-free to contact the PI via the following address:-

Tilaye Arega Moges (B.Pharm, MSc in clinical pharmacy)

Lecturer, School of Pharmacy, Department of clinical pharmacy

College of Health Sciences, Debre Tabor University

P.O. Box 272

Debre Tabor, Ethiopia

e-mail: tilayearega@gmail.com

Written Informed consent form

By understanding the foregoing information, I'm giving my consent to participate in a study entitled "**Determinants of Medication Non-Adherence among Patients with Chronic Disease s at Community Pharmacy Settings in South Gondar Zone, Northwest Ethiopia: A Multice nter Cross-Sectional Study**". I have read or it has been read to me the foregoing information about the nature of the study, benefits, voluntary participation, confidentiality and withdrawal from the study at any time without any harm. I have had the opportunity to ask questions about it and any questions I have been asked have been answered to my satisfaction. I consent voluntarily to be a participant in this study.

Are you willing to participate in this study?

a. Yes, I agree

b. No, I don't agree

Code of the study participant___________________

Signature of the study Participant ____________________

Date ____________________________

.

Thank you very much for your willingness to participate honestly and cooperatively

If yes, continue the interview; code: ___________

Annex II: Data collection tool/ Questionnaire (English version)

This tool was used to assess **"Determinants of medication non-adherence among patients with chronic diseases at community pharmacy settings in South Gondar, Northwest Ethiopia: A multicenter cross-sectional study"**

Dear data collector(s),

First of all I would like to thank you for your willingness to accept my request and to participate as a data collector in this study which will surely be of great significance in contributing to determine the medication non-adherence level and its determinants among patients with chronic diseases at community pharmacy settings in South Gondar, Northwest Ethiopia. This data will then enable the concerned bodies to emphasize on the identified gaps and the associated factors to formulate and implement scheduled on job training for healthcare professionals, chronic patient medication use evaluation, and community pharmacy engagement and on job training of pharmacists in chronic disease management as a part of health care quality measurement in turn to boost patient safety and reduce suffering and enhance excellence in patient care for the future. Finally, I would like to greatly acknowledge, in advance, your indispensible roles in maintaining the quality of this data honestly and effective output of the present study. I promise you to be timely respond whenever you face challenges during the data collection period.

The principal investigator!

Data collection tool/questionnaire

Assessing Non-Adherence to Chronic Medications among Patients with chronic diseases Attending Community Pharmacies at South Gondar Zone, Northwest Ethiopia: A Multicenter Cross-Sectional Study

Part I: Socio-demographic characteristics of the study participants.

1.1 Community pharmacy location

a. Gayint
b. Debre tabor
c. Addiszemen
d. Woreta
e. Andebet
f. Este
g. Semeda

1.2 Gender a. male b. female

1.3 Age a. ≥65 b. <65

1.4 Weight in kg:______

1.5 BMI:________

1.6 Residence a. Rural b. Urban

1.7 Religion: a. Orthodox b. Muslim c. Protestant d. Other; specify:________

1.8 Marital status: a. Single b. Married c. Divorced d. Widowed

1.9 Educational status: a. Can't read and write b. Non–formal education c. Primary school (1-8) d. Secondary school (9-12) e. Tertiary education (diploma and above)

1.10 Job status: a. Employed b. Unemployed c. Other Specify: _______________

1.11 Source of medication fee: a. free b. payment

1.12 medication management: a. autonomous/self b. family/relatives

1.13 ADR: a. yes b. no

1.14 Regular Physical activity/exercise: a. yes b. no

1.15 Smoking habit: a. yes b. no

1.16 Alcohol habit: a. yes b. no

1.17 Herbal Medicine Use: a. Yes b. No

1.18 Have you experienced side effects by chronic medications:

a. Yes b. No

1.19 Chronic disease duration (in years):________

1.20 Charlson comorbidity index (CCI):__________

1.21 Do you receive medication counseling for all chronic medications:

a. yes b. no

1.22 Monthly income (Ethiopian birr): a. < 1500 b. 1500-4000 c. 4000-5000 d. >5000

1.23 Good medication knowledge: a. Yes b. No

Part II: Clinical and medication related characteristics

2.1 Chief compliant/current assessment :_________________________

2.2 HPI:____

2.3 Other chronic conditions (other than the current assessment)

a. ____

b. ____

c. ____

d. ____

e. ____

2.4 Past medication history:____

____

____

____

2.5 Number of medications consumed:__________________

a. 1–4 medicines
b. ≥ 5 medicines

2.6 Duration of Medication use

a. <one year
b. ≥one year

2.7 Medications used by study participants (current medications); Name, Dose, Route, Frequency, and Duration of therapy is must.

I. ____
II. ____
III. ____
IV. ____

V. ____

VI. ____

VII. ____

VIII. ____

2.8 Therapeutic group of medicines (According to the ATC classification system)

☐ A:alimentary tract and metabolism	☐ L: Antineoplastic and immune-modulating agents
☐ B:blood and blood-forming organs	☐ M: muscular-skeletal system
☐ C:cardiovascular system	☐ N: nervous system
☐ D:dermatologicals	☐ P: Anti-parasitic products
☐ G: genitourinary system and sex hormones	☐ R: respiratory system
☐ H: Systemic hormonal preparations, excluding sex hormones and Insulin	☐ S: sensory organs
☐ J: Anti-infective for systemic use	☐ V: Various

Part III: Medication knowledge among chronic disease patients.

Question	Yes	No
3.1 Can you list the name of medications you are currently taking?		
3.2 Can you tell me why you are taking this medication?		
3.3 Do you know how to take medication?		
3.4 Do you know when to take your medicine?		
3.5 Do you know the possible side effects of your medicines?		
3.6 Do you know what to do if your medication side effects occur?		
3.7 Do you know what to do if you miss a dose of your medicines?		

For question 3.2: Write reasoning of study participants in general terms:

____

____

____

____

____

____

____

____

Part IV: Assessment of medication adherence of chronic disease patients attending community pharmacies

The Adherence in Chronic Diseases Scale (ACDS) to assess medication adherence of chronic disease patients attending community pharmacies at South Gondar zone, Northwest, Ethiopia.

Instruction: - Choose the correct those represents you and encircle it.

4.1. Do you always remember to take all your medications according to your doctor's instructions?

A. Always

B. Almost always

C. Sometimes

D. Hardly ever

E. Never

4.2. Do you happen to change the dosing of your medications without prior consultation with your doctor?

A. Never

B. only occasionally

C. Sometimes

D. Frequently

E. I do not adhere to my doctor's recommendations at all

4.3. Do you adjust the dosing of your medications according to how you feel?

A. No, I strictly follow the prescribed dosing, no matter how I feel

B. Yes, I reduce the dosage of some medications when I feel good

C. Yes, I skip doses of some medications when I feel good

D. Yes, I temporarily discontinue some medications when I feel good

E. Yes, I discontinue all medications when I feel good

4.4. On the appearance of medication-related side effects (e.g. stomach pain, liver pain, rash, lack of appetite, edema):

A. I seek medical attention instantly

B. I reduce the dosage of the medication and attempt to expedite the elective appointment with my doctor

C. I discontinue the medication and attempt to expedite the elective appointment with my doctor

D. I discontinue the medication and wait for the next elective appointment with my doctor

E. I discontinue all my medications and wait for the next elective appointment with my doctor

4.5. Do you find all your medications necessary for your health?

A. Yes, I do

B. I find most of my medications to be beneficial for my health

C. I find only some of my medications to be beneficial for my health

D. I find some of my medications to be beneficial for my health, while the others to be harmful for me

E. I find the majority of my long-term medications to be harmful for me

4.6. Does your doctor inquire about medication-related problems that you might possibly experience?

A. Yes, on every appointment

B. Yes, he/she usually does

C. Yes, but only sometimes

D. Yes, but only occasionally

E. No, never

4.7. Do you tell truth when asked by your doctor about medication-related problems?

A. Yes, always

B. Almost always

C. I try to be honest, but sometimes it is hard to admit to non-compliance with doctor's recommendations

D. Sometimes yes, another time no

E. No, I don't. I find it my own private business

NB:

Results range from 0–28 points

Score

A =4	C = 2	E = 0
B =3	D =1	

Total score < 21 points reflects Low adherence

Total score 21–26 points reflects medium adherence

Total score > 26 points reflects high adherence

የማህበረሰብ አቀፍ መድኃኒት ቤት (COMMUNITY PHARMACY) ተጠቃሚዎች የመድኃኒት አወሳሰድ ታማኝነት (medication adherence) ለመገምገም ያለው የመጠን ልኬት

መመሪያ፡- እርስዎን የሚወክለውን ትክክለኛውን ይምረጡ እና ያክብቡ ።

1. በሐኪምዎ መመሪያ መሠረት ሁሉንም መድሃኒቶችዎን መውሰድዎን ሁልጊዜ ያስታውሳሉ?

ሀ. ሁልጊዜ

ለ. ሁልጊዜ ማለት ይቻላል

ሐ. አንዳንድ ጊዜ

መ. በጭራሽ

ሠ. በጭራሽ አላደርገውም

2. ከሐኪምዎ ጋር ያለ ቅድመ ምክክር ያለዎትን የመድኃኒት ልክ /መጠን / ይለውጣሉ

ሀ. በጭራሽ

ለ. አልፎ፤አልፎ ብቻ

ሐ. አንዳንድ ጊዜ

መ. በተደጋጋሚ

ሠ. በጭራሽ የዶክተሮቼን ምክር አላከብርም

3. በሚሰማዎት ስሜት መሠረት የመድኃኒቶችዎን ልክ መጠን ያስተካክላሉ?

ሀ. አይ ፣ እኔ ምንም ያህል ቢሰማኝም የታዘዘልኝን መድሃኒት በጥብቅ እከተላለሁ

ለ. አዎ ፣ ጥሩ ስሜት ሲሰማኝ የአንዳንድ መድኃኒቶችን መጠን እቀንሳለሁ

ሐ. አዎ ፣ ጥሩ ስሜት ሲሰማኝ የአንዳንድ መድኃኒቶችን መጠን እዘላለሁ

መ. አዎ ጥሩ ስሜት ሲሰማኝ አንዳንድ መድሃኒቶችን ለጊዜው አቋርጣለሁ

ሠ. አዎ ፣ ጥሩ ስሜት ሲሰማኝ ሁሉንም መድሃኒቶች አቋርጣለሁ

4. ከመድኃኒት ጋር የተዛመዱ የጎንዮሽ ጉዳቶች ሲከሰቱ (ለምሳሌ የሆድ ህመም ፣ የጉበት ህመም ፣ ሽፍታ ፣ የምግብ ፍላጎት መቀነስ ፣ እብጠት)

ሀ. ወዲያውኑ የሕክምና እርዳታ እፈልጋለሁ

ለ. የመድኃኒቱን መጠን እቀንሳለሁ እና ከሐኪሜ ጋር የተመረጠውን ቀጠሮ ለማፋጠን እሞክራለሁ

ሐ. መድሃኒቱን አቋርጬ እና ከሐኪሜ ጋር የተመረጠውን ቀጠሮ ለማፋጠን እሞክራለሁ

መ. መድሃኒቱን አቋርጬ ከሐኪሜ ጋር የሚቀጥለውን የምርጫ ቀጠሮ እጠብቃለሁ

ሠ. ሁሉንም መድኃኒቶቼን አቋርጬ ከሐኪሜ ጋር የሚቀጥለውን የምርጫ ቀጠሮ እጠብቃለሁ

5. ሁሉንም መድሃኒቶችዎን ለጤንነትዎ አስፈላጊ ሆነው ያገኙቸዋል?

ሀ. አዎ ፣ አስፈላጊ ሆነው አግኝቼቸዋለሁ

ለ. አብዛኛዎቹ መድሃኒቶቼ ለጤንነቴ ጠቃሚ ሆነው አግኝቼቸዋለሁ

ሐ. ለጤንነቴ ጠቃሚ የሆኑ አንዳንድ መድኃኒቶቼን ብቻ አገኛለሁ

መ. አንዳንድ መድሃኒቶቼ ለጤንነቴ ጠቃሚ ሲሆኑ ሌሎቹ ደግሞ ለእኔ ጎጂ ሆነው አግኝቼቸዋለሁ

ሠ. አብዛኛዎቹ የረጅም ጊዜ መድሃኒቶቼ ለእኔ ጎጂ ሆነው አግኝቼቸዋለሁ

6. ሐኪምዎ ምናልባት ሊያጋጥሙዎት ከሚችሉት መድኃኒቶች ጋር ስለሚዛመዱ ችግሮች ይጠይቃል/ትጠይቃለች

ሀ. አዎ ፣ በእያንዳንዱ ቀጠሮ ላይ	ሐ. አዎ ፣ ግን አንዳንድ ጊዜ ብቻ
ለ. አዎ፣ እሱ/እሷ ብዙውን ጊዜ	መ. አዎ ፣ ግን አልፎ፤አልፎ
ይጠይቃል/ትጠይቃለች	ሠ. አይ፣በጭራሽ

7. ከ መድኃኒት ጋር ስለሚዛመዱ ችግሮች በሐኪምዎ ሲጠየቁ እውነቱን ይናገራሉ?

ሀ. አዎ ፣ ሁልጊዜ

ለ. ሁልጊዜ ማለት ይቻላል

ሐ. ሐቀኛ ለመሆን እሞክራለሁ ፣ ግን አንዳንድ ጊዜ የዶክተሮችን ምክር አለመከተልን በነሱ ፊት ማመን በጣም ከባድ ነው

መ. አንዳንድ ጊዜ አዎ፣ሌላ ጊዜ የለም

ሠ. አይ, እኔ አላደርግም።የራሴ የግል ጉዳይ ስለሆነ

እናመሰግናለን።

Unique ID/Code _______________	*Name of the data collector:* ____________
Date: ____________	*Comment:* ____________
Signature: ____________	*Date:* ____________
Checked by: ____________	*Sig.:*____________

Thank You for your valuable participation!
